# Detection of influenza A virus from live-bird market poultry swab samples in China by a pan-IAV, one-step reverse-transcription FRET-PCR

**DOI:** 10.1038/srep30015

**Published:** 2016-07-22

**Authors:** Lu Luan, Zhihao Sun, Bernhard Kaltenboeck, Ke Huang, Min Li, Daxin Peng, Xiulong Xu, Jianqiang Ye, Jing Li, Weina Guo, Chengming Wang

**Affiliations:** 1 Jiangsu Co-innovation Center for the Prevention and Control of Important Animal Infectious Diseases and Zoonoses, College of Veterinary Medicine, Yangzhou University, Yangzhou, Jiangsu Province, P. R. China; 2 College of Veterinary Medicine, Auburn University, Auburn, Alabama, USA

## Abstract

The persistent public health threat of animal to human transmission of influenza A virus (IAV) has stimulated interest in rapid and accurate detection of all IAV subtypes in clinical specimens of animal origin. In this study, a new set of primers and probes was designed for one-step pan-IAV reverse-transcription fluorescence resonance energy transfer (FRET)-PCR. The detection limit of one-step pan-IAV RT FRET-PCR was 10 copies of the matrix gene per reaction, and proved to be equivalent or superior to virus isolation in detecting nine IAV subtypes. Application of the pan-IAV RT FRET-PCR to oral-pharyngeal and cloacal swab specimens collected from healthy poultry in 34 live bird markets in 24 provinces of China revealed that 9.2% of the animals (169/1,839) or 6.3% of their oral-pharyngeal or cloacal swabs (233/3,678) were positive for IAV, and 56.8% of IAV-positive samples were of the H9N2 subtype. Paralleling detection of IAV in H9N2-infected SPF chickens and chickens from LBM showed that pan-IAV FRET-PCR had a higher detection limit than virus isolation in eggs while the results by FRET-PCR and virus isolation overall matched. It is expected that this strategy can be useful for facile surveillance for IAV in clinical samples from a variety of sources.

Live-bird markets (LBMs) in villages, small-community and even urban areas are essential for marketing poultry in many regions of the world, including China, and they bring together a mixture of bird species that are commonly produced by multiple suppliers to meet the preferences of their customers^1^,^2^,^3^. These birds are commonly held in cages and baskets, or strapped for sale. The birds typically do not remain in the market continuously and are often returned to the supplier if not sold.

The presence of various bird species including chicken, duck, goose and pigeon from multiple suppliers and reselling of the unsold birds in LBMs next day make LBMs a potential source for influenza A virus^2^,^3^. IAV transmission dynamics via LBMs have driven the emergence of highly pathogenic avian influenza H5N1 and other subtypes in several countries^2^,^3^,^4^,^5^,^6^,^7^,^8^. Therefore, it is imperative and critical to establish a convenient and sensitive tool to monitor IAV in LBMs. Traditionally, the gold standard for IAV detection involves virus replication in eggs or tissue culture followed by HA inhibition and NA inhibition assays^9^,^10^,^11^. While the virus propagation in egg and inhibition assays are laborious and not very sensitive, many powerful reverse transcription-PCR and real-time RT-PCR assays have been successfully established for IAV detection^10^,^11^,^12^,^13^,^14^,^15^,^16^,^17^,^18^,^19^,^20^. However, to the best of our knowledge, there is no real-time one-step RT-PCR test which can conveniently detect most IAV subtypes in clinical samples. Therefore, there is a need for a simple and fast test that detects most subtypes of influenza A viruses of avian and porcine origin in LBM or wet markets (mammals are also marketed in LBMs).

The aim of this study was to develop a rapid and sensitive one-step RT-PCR which has the universal ability to detect the presence of many phenotypically and genotypically diverse IAV strains in clinical samples. To this end, we applied the pan-IAV one-step RT Fluorescence Resonance Energy Transfer (FRET)-PCR to oral-pharyngeal and cloacal swabs obtained from poultry in LBMs of 24 provinces of China.

## Results

### Establishment of the one-step pan-IAV RT FRET-PCR

**Figure 1:**
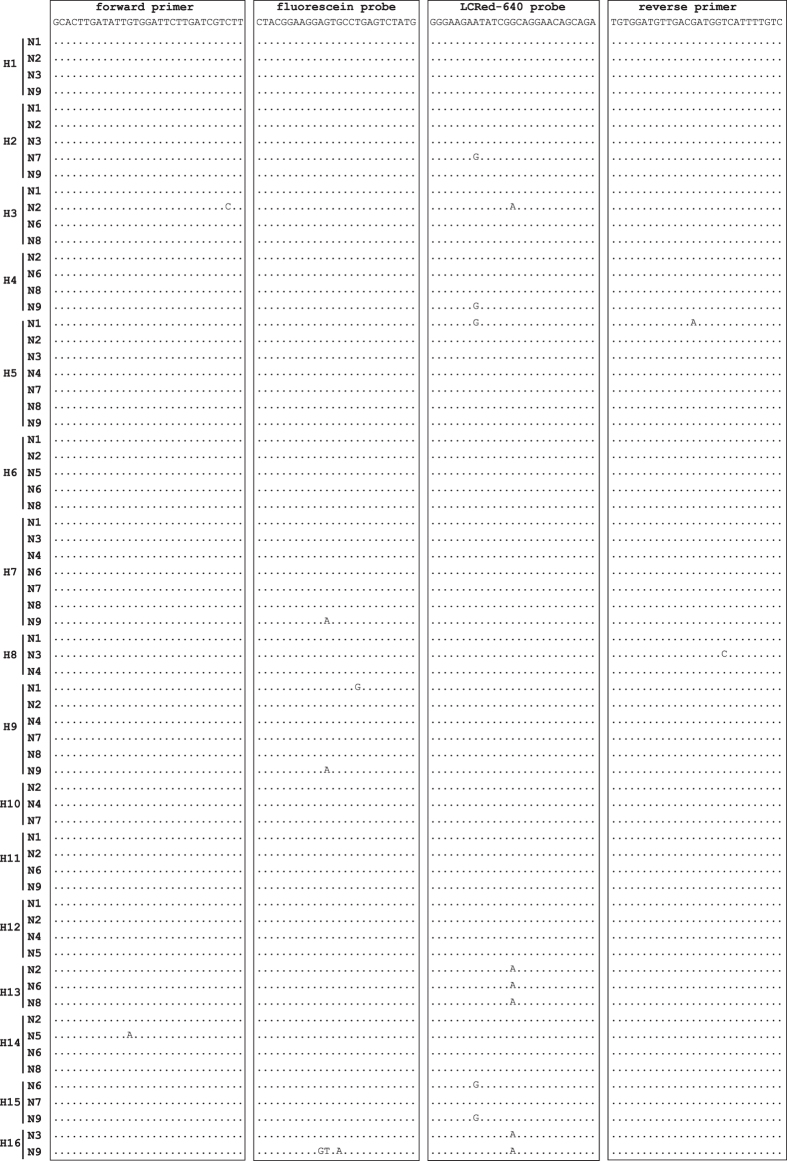
Alignment of the primers and probes of the pan-IAV PCR with the IAV M gene sequences. The sequences of the forward/reverse primers and the fluorescein/LCRed 640 probes are shown at the top of the boxes. The forward primer and two probes were used as the demonstrated sequences without gaps while the reverse primer was used as an antisense oligonucleotide. The 6-FAM label was attached directly to the 3-terminal nucleotide of the fluorescein probe, and the LCRed 640 fluorescein label was added via a linker to the 5′-end of the LCRed 640 probe. Dots indicate nucleotides identical to the primers and probes. The designed oligonucleotides show minimal mismatches with influenza A virus (0 mismatch with 52 IAV subtypes, 1 mismatch with 13 subtypes, 2 mismatches with 2 subtypes and 4 mismatches with 1 subtypes).

The primers and probes we designed were identical with 52 IAV subtypes, had 1 mismatch with 13 subtypes, 2 mismatches with 2 subtypes and 4 mismatches with 1 subtype (Fig. 1). The nucleotide BLAST for individual primers and probes detected a total of 28,206 homologous entries, encompassing 122 IAV subtypes (Table S1). To confirm the results of BLAST partially, the pan-IAV RT FRET-PCR we developed was found to specifically detect RNAs of all 9 tested IAV subtypes (H1N1, H3N3, H4N6, H5N1, H6N1, H7N9, H9N2, H10N8 and H11N2).

**Figure 2:**
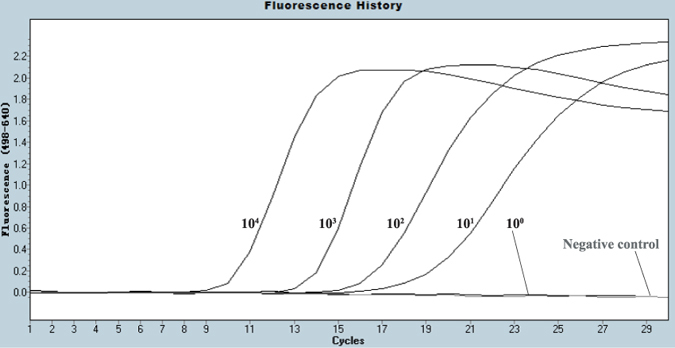
PCR amplification curves of IAV standards. The IAV H1N1-derived standards were logarithmically diluted (10^4^ to 10^0^ copies/reaction) for amplification by pan-IAV RT-qPCR. The 10^4^ copies amplification curve crosses the fluorescence threshold at approximately cycle 9, while the 10^1^ copy cross at approximately cycle 16. In contrast, the 10^0^ copy does not cross the threshold and is indistinguishable from the negative control.

**Figure 3:**
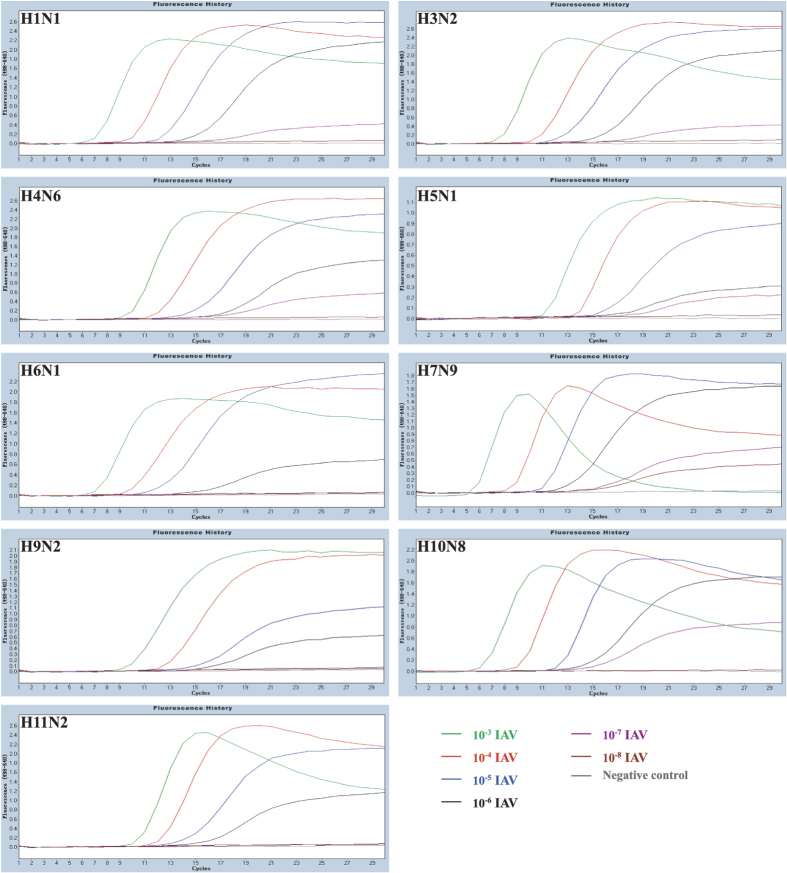
PCR amplification curves of nine serially-diluted IAV subtypes. Logarithmically diluted murine lung homogenates inoculated with nine IAV subtypes were (10^−3^ green line, 10^−4^ red, 10^−5^ blue, 10^−6^ black, 10^−7^ purple and 10^−8^ purplish red) were amplified by the pan-IAV RT FRET-PCR established in this study. The detection sensitivity was the 10^−6^ dilution for IAV of H6N1, H9N2 and H11N2; 10^−7^ dilution for H1N1, H3N3, H4N6, H5N1, and H10N8; 10^−8^ dilution for H7N9.

The detection limit of the one-step pan-IAV RT FRET-PCR was 10 copies per reaction based on H1N1-derived standards (Fig. 2). Testing on the 10-fold dilutions of 9 IAV subtypes showed that the detection limit of the one-step pan-IAV RT FRET-PCR was equivalent or superior to that of the EID_50_ measurement (Table 1, Fig. 3).

### Prevalence of IAV in live-bird market poultry in China

The one-step pan-IAV RT FRET-PCR detected that 9.2% of poultry (169/1,839) from Chinese LBMs was positive for IAV (Table 2). IAV was detected in the poultry swabs from 9 of 24 provinces tested, and the positivity varied from 1.7% to 45.2% in different provinces (Table 2). Chickens were most commonly positive (10.4%; 158/1,521), followed by ducks (5.5%; 7/127), pigeons (4.4%; 4/91) and geese (0%; n = 100).

Overall, 6.3% of the swabs (233/3,678) were positive for IAV in this study. IAV positivity of oral-pharyngeal swabs (7.5%, 138/1,839) was significantly higher than of cloacal swabs (5.2%, 95/1,839) (P < 10^−4^). In a similar trend, the copy number/swab was also significantly higher in oral-pharyngeal swabs than in cloacal swabs (10^6.07^ vs. 10^5.54^; P = 0.0001). Among the IAV-positive poultry, 27.8% were positive in both oral-pharyngeal and cloacal swabs while 47.9% were only positive in oral-pharyngeal swabs, and 24.3% were only positive in cloacal swabs.

The genus-specific polymerase acidic protein (PA) gene-based PCR with a long amplicon (575 bp) was able to amplify all 9 IAV subtypes. RNA from 48 of 50 IAV-positive swabs determined by pan-IAV RT FRET-PCR and with > 20,000 IAV copy/swab was successfully amplified by the generic PA-gene PCR, and verified by gel electrophoresis and genomic sequencing.

The neuraminidase (NA) gene-based H9N2-specific PCR revealed that 56.8% of IAV-positive samples determined by pan-IAV RT-qPCR (96/169) belonged to the H9N2 subtype. Subtype H9N2 was detected in swabs from chickens and ducks in 7 of the 9 IAV-positive provinces in this study (Table 2). The data for Highly pathogenic avian influenza virus will be presented in another paper.

### Comparative IAV detection by pan-IAV FRET-PCR and virus isolation

Oral-pharyngeal and cloacal swabs artificially spiked with H9N2 IAV were used for comparative IAV detection by pan-IAV FRET-PCR and virus isolation in eggs. The detection limit for H9N2 IAV was 10^−6^ dilution by pan-IAV FRET-PCR which was 10 times higher than the 10^−5^ dilution by virus isolation.

**Figure 4:**
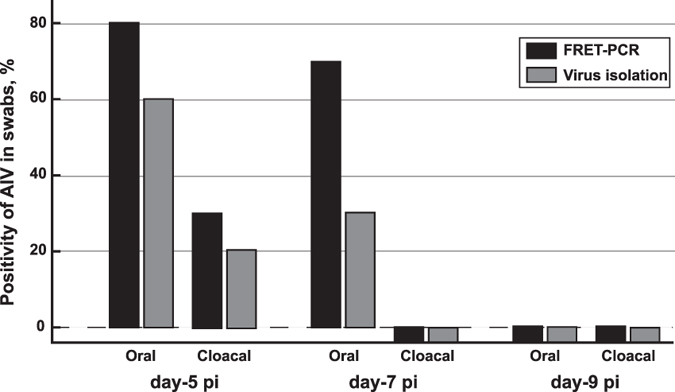
IAV detection in oral-pharyngeal and cloacal swabs of H9N2-infected SPF chickens by pan-IAV FRET-PCR and virus isolation. Three-week-old specific pathogen-free (SPF) White Leghorn chickens (n = 10) were inoculated intranasally with 10^6^ EID_50_ of IAV H9N2 in a 0.2 ml volume. Oral-pharyngeal and cloacal swabs were collected from chickens at 5, 7 and 9 dpi, and resuspended in 1 ml PBS for total nucleic acids extraction followed by FRET-PCR and for virus isolation in the allantoic cavities of 10-day-old embryonated chicken eggs. The pan-IAV FRET-PCR determined the presence of IAV RNA in 8 oral-pharyngeal swabs and 3 cloacal swabs at day-5 pi, and 7 oral-pharyngeal swabs at day-7 pi. In a contrast, virus isolation for H9N2 was positive in 6 oral-pharyngeal swabs and 2 cloacal swabs of chickens at day-5 pi, and 3 oral-pharyngeal swabs at day-7 pi. IAV was not detected in the cloacal swabs of day-7 pi and both types of swabs of day-9 pi by the pan-IAV FRET-PCR and virus isolation. Swabs determined to be positive by virus isolation were all positive detected by the pan-IAV FRET-PCR in animal experiment.

**Figure 5:**
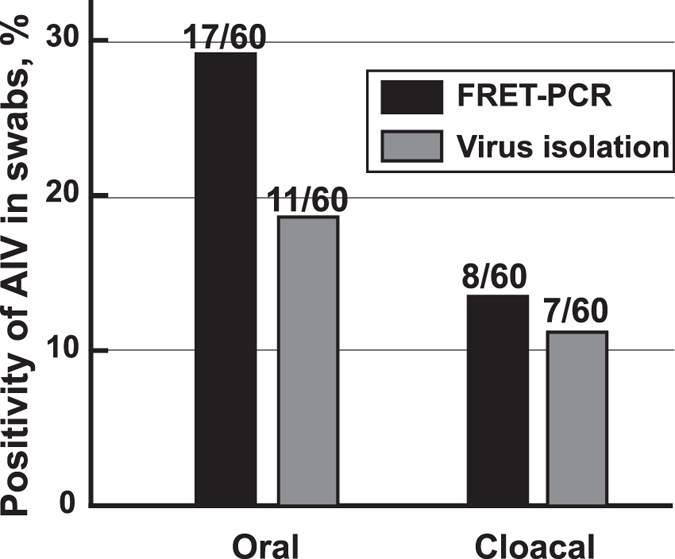
Detection of IAV in oral-pharyngeal and cloacal swabs of chickens from a live-bird market by pan-IAV FRET-PCR and virus isolation. In May of 2016, oral-pharyngeal swabs and cloacal swabs were collected from apparently healthy chickens (n = 60) into 1 ml antibiotics-containing PBS (penicillin 10,000 unit/mL, streptomycin 10 mg/mL, gentamycin 250 μg/mL, kanamycin, 250 μg/mL) for IAV detection by pan-IAV FRET-PCR and virus isolation. The pan-IAV FRET-PCR identified 22 positive chickens (36.7%) with IAV in 17 oral-pharyngeal swabs and 8 cloacal swabs. In contrast, virus isolation determined 17 IAV-positive chickens (28.3%) with 11 positive oral-pharyngeal swabs and 7 positive cloacal swabs. While chickens determined to be positive in cloacal swabs were all positive in oral-pharyngeal swabs by pan-IAV FRET-PCR, one chicken was positive in cloacal swabs but negative in oral-pharyngeal swab by virus isolation. Chickens determined to be positive by virus isolation were all positive based on the pan-IAV FRET-PCR.

Comparative IAV detection in oral-pharyngeal and cloacal swabs of H9N2-infected SPF chickens by pan-IAV FRET-PCR and virus isolation demonstrated that FRET-PCR is more sensitive than virus isolation (Fig. 4). In a similar trend, FRET-PCR determined that 36.7% of the assayed chickens was IAV positive, being higher than the 28.3% positivity by virus isolation (Fig. 5). Virus isolation and identification showed that 44.4% of 17 IAV-positive samples belonged to H9 subtypes while 6 of them carried one IAV subtype, 8 with dual subtypes and 3 with 3 subtypes. However, standard Sanger sequencing of the 169-bp amplicon from the pan-IAV FRET-PCR generally showed high similarities to multiple IAV subtypes and cannot determine the IAV serovar or co-infections of the detected samples.

## Discussion

PCR-based strategies have been successfully used for detection of many clinically relevant pathogens including IAV^11^,^12^,^21^,^22^,^23^,^24^. The sensitivities and specificities of PCR-based methods are critically determined by the choice of the sequences for primers and probes^23^,^24^. The sequences for the primers and/or probes which were described earlier for IAV detection may be appropriate for some IAV subtypes, or strains only circulating in humans or poultry, but have many mismatches to IAV strains found in other animals^11^,^12^,^13^,^14^,^15^,^16^,^17^,^18^,^19^,^20^. In this study, we used the extensive sequence information available for IAV in GenBank to design a pan-IAV primer and probe set. The designed PCR amplified all nine IAV subtypes tested, and showed detection sensitivity and specificity equal or superior to the standard EID_50_ measurement. While the BLASTN search indicated that the primers and probes were highly similar to 23,026 IAV sequences in GenBank, covering 122 IAV subtypes, the pan-IAV FRET-PCR was confirmed to amplify all nine tested IAV subtypes with an equivalent or superior detection limit than the EID_50_ measurement.

The current methodology in surveillance of animal-origin IAV in China includes mainly virus inoculation followed by hemagglutination and/or PCR-based subtyping^11^,^12^,^13^,^25^, and this approach is laborious, takes approximately 7 days and requires rapid transportation to keep IAV viable in the collected specimens for virus isolation. In contrast, the established method in this study can reliably preserve the collected samples at room temperature free of biohazard and the definite conclusion of the IAV status in clinical samples can be made within 4 hours from extraction of total nucleic acids. The established method in this study can be used to conveniently and accurately monitor the presence of IAV in a wide range of hosts, regions and time points. One disadvantage of the pan-IAV FRET-PCR established in this study is that this assay cannot determine the IAV subtype, or co-infections in samples. In addition, viral RNA tends to degrade during improper storage and transport of specimens which may leads to false negative results. Therefore, it is of great importance that samples are well preserved, and positive and negative controls need to be included to determine cutoff value during performing RT-PCR for IAV detection. Ideally, the positive samples determined by this pan-IAV FRET PCR would be further verified by virus isolation and identification in eggs. Combination of this pan-IAV FRET-PCR with virus isolation provides a fast and reliable approach for IAV detection in clinical samples.

To the best of our knowledge, this is the first one-step pan-IAV real-time reverse-transcription PCR which has been validated by amplifying nine IAV However, standard Sanger sequencing being validated with virus isolation, and was used to detect IAV in poultry swabs under clinical diagnostic conditions. Because of the highly matched primers and probes to most IAV subtypes, it is expected that this pan-IAV detection strategy will be suitable for convenient surveillance of the IAV in clinical samples from a variety of sources such as LBMs.

## Materials and Methods

### Ethics statement

This study was reviewed and approved by the Institutional Animal Care and Use Committee of Yangzhou University College of Veterinary Medicine, and was carried out in accordance with the approved guidelines^25^.

### IAV strains

Nine IAV strains (H1N1, H3N3, H4N6, H5N1, H6N1, H7N9, H9N2, H10N8 and H11N2) isolated from chickens and ducks in Chinese LBMs between 2005–2014 were kindly provided by the Key Laboratory for Avian Preventive Medicine, Ministry of Education, Yangzhou University, Jiangsu, China (Table 1). In addition, Class I and Class II Newcastle disease viruses, infectious bursal disease virus, infectious bronchitis virus and infectious laryngotracheitis virus were also provided by Yangzhou University to serve as negative control for this assay.

### Animal experiments

#### Artificially inoculated oral-pharyngeal and cloacal swabs

One-day-old SPF AA broiler chickens (n = 10) purchased from Sandeli Animal Husbandry Development Co., Ltd (Zhenjiang, Jiangsu, China) were housed in a containment level 2 facility with free access to food and water. After one week, oral-pharyngeal and cloacal swabs were collected from each of the broilers into tubes containing a mixture of 200 μl 10^6^ EID_50_ of IAV H9N2 and an equal volume of 1 ×PBS which was then 10-fold serially diluted. The 100 μl serially-diluted aliquots were used for extraction of total nucleic acids as described below while another 100 μl was used to inoculate embryonated eggs via the allantoic cavity for virus isolation.

#### H9N2-infected SPF chickens

Three-week-old specific pathogen-free (SPF) White Leghorn chickens (n = 10) were inoculated intranasally with 10^6^ EID_50_ of IAV H9N2 in a 0.2 ml volume. Chickens were hatched out from SPF embryonated chicken eggs purchased from Beijing Experimental Animal Center, Beijing, China. Oral-pharyngeal and cloacal swabs were collected from chickens at 5, 7 and 9 dpi, and resuspended in 1 ml PBS for total nucleic acids extraction followed by FRET-PCR and for virus isolation in the allantoic cavities of 10-day-old embryonated chicken eggs (Beijing Experimental Animal Center, Beijing, China).

#### Virus isolation and identification

In May 2016, oral-pharyngeal swabs and cloacal swabs were collected from chickens (n = 60) in a LBM of Yangzhou for IAV detection by generic FRET-PCR and virus isolation. The swabs were collected into 1 ml antibiotics-containing PBS (penicillin 10,000 unit/mL, streptomycin 10 mg/mL, gentamycin 250 μg/mL, kanamycin, 250 μg/mL) which were used for extraction of total nucleic acids followed by FRET-PCR and for virus isolation in the allantoic cavities of 10-day-old embryonated chicken eggs. After approximately 1 h incubation at room temperature, 100 μl medium from each sample was inoculated into the allantoic cavity of 10-day-old SPF embryonated chicken egg. After 3 days of incubation at 35 °C, the presence of hemagglutinating agents was determined by the hemagglutination assay using 0.5% chicken erythrocytes. The HA (H1-H15) subtypes of the IAV were identified with a hemagglutination inhibition test (antiserum against the H1-H15 subtype was supplied by the China National Avian Influenza Reference Laboratory, Haerbin, China). Experiments using infectious viruses were conducted in biosafety level-2 laboratory facilities.

#### Oral-pharyngeal and cloacal swab samples

Oral and cloacal swabs were used that had been collected from apparently healthy poultry (n = 1,839) in 37 LBMs of 24 provinces in China between December 2013 and February 2014 for molecular epidemiology of *Chlamydia* spp^26^. The birds were randomly chosen for collecting swabs with the permission from the sellers or owners. In general, we tried to collect birds of various species and not to collect swabs from more than 80 birds in one LBM. One oral-pharyngeal swab and one cloacal swab were collected from each bird, and the poultry examined in this study were primarily from local farmers other than from large commercial farms. The swabs were immediately placed in sterile tubes containing 400 μl DNA/RNA stabilization buffer (Roche Molecular Biochemicals, Indianapolis, IN, USA), a highly denaturing guanidine-isothiocyanate-containing buffer, to preserve the nucleic acids and kill microbes, and then stored at −80 °C until total nucleic acids were extracted.

#### Extraction of total nucleic acids

The High-Pure PCR Template Preparation Kit (Roche Molecular Biochemicals, Indianapolis, IN, USA) was used to extract total nucleic acids from oral-pharyngeal and cloacal swabs according to the manufacturer’s instructions and as described before^22^. The extracted nucleic acids were eluted in 200 μl elution buffer for each swab and preserved at −80 °C until PCR described below.

### Development of a universal IAV one-step reverse-transcription fluorescence resonance energy transfer PCR (pan-IAV RT FRET-PCR)

#### Primers and probes

Nucleic acid sequences of sixty-eight representative IAV serovars including subtype combinations (H1-H16 and N1-N9) were obtained from GenBank (Table S2). The Clustal Multiple Alignment Algorithm was used to align all sequences, and a highly conserved region of the matrix gene was selected as PCR target for amplification of all IAV subtypes (Fig. 1), using these primers and probes: forward primer 5′-GCACTTGATATTGTGGATTCTTGATCGTCTT-3′, reverse primer 5′-GACAAAATGACCATCGTCAACATCCACA-3′, fluorescein probe 5′-CTACGGAAGGAGTGCCTGAGTCTATG-(6-FAM)-3′ and LightCycler Red 640 probe 5′-LCRed640-GGGAAGAATATCGGCAGGAACAGCAGA-phosphate-3′.

#### Nucleotide BLAST search of primers and probes

The primers and probes designed for this study were individually blasted against all IAV sequences in GenBank under the option of Megablast (searching for highly similar sequences). The BLASTN entries obtained for each primer/probe on each IAV subtypes (H1-H16 and N1-N9) were recorded (Table S2), and the lowest number of the obtained entries for each of these four oligonucleotides was assumed as the number of strains of an IAV subtype which can be amplified and detected by the pan-IAV RT-FRET PCR. Subtypes for which one of the primers or probes did not match were assumed non-detectable or not existing as shown in Table 3 and Table S1.

#### Thermal cycling

High-resolution melting curve analysis following PCR was performed on a Roche Light-Cycler 480-II platform as described before^27^,^28^. The one-step RT PCR consisted of one cycle at a 30 min reverse-transcription step at 55 °C followed by 18 high-stringency step-down thermal cycles, 40 low-stringency fluorescence acquisition cycles, and melting curve determination between 38 °C and 85 °C. The cycling parameters for FRET-qPCR were 1 × 30 min at 55 °C; 6 × 1 sec at 95 °C, 12 sec at 72 °C, 30 sec at 72 °C; 9 × 1 sec at 95 °C, 12 sec at 70 °C, 30 sec at 72 °C; 3 × 1 sec at 95 °C, 12 sec at 68 °C, 30 sec at 72 °C; 40 × 1 sec at 95 °C, 8 sec at 56 °C and fluorescence acquisition, 30 sec at 67 °C, 30 sec at 72 °C; and 1 × 38 °C to 85 °C in 2.2 °C increments with continuous fluorescence acquisition.

#### Detection limit

For use as quantitative standards, the products of the universal IAV RT FRET-PCR on H1N1 virus were gel purified using a QIAquick Gel Extraction Kit (Qiagen, Valencia, CA, USA). An aliquot of the purified DNA was sequenced for confirmation at GenScript (Nanjing, Jiangsu, China) and the remainder was quantified in a PicoGreen assay (Invitrogen). After using the molecular mass of the matrix gene amplification target to calculate the molarity of the solution, dilutions were made to give solutions containing 10,000, 1,000, 100, 10, 1 gene copies per reaction (Fig. 2). These were amplified by pan-IAV RT FRET-PCR in triplicates to determine the detection limit of the PCR.

The detection limit of the one-step pan-IAV RT FRET-PCR was also determined by comparing the amplification of 10-fold serially diluted RNAs of 9 IAV subtypes to the quantification by the EID_50_ and hemagglutination assays (Table 1, Fig. 3). Each of the nine IAV subtypes contained in lung homogenates of challenged mice was 10-fold serially diluted in 1 ×PBS. A 100 μl aliquot was used for extraction of total nucleic acids as described above, while another 100 μl was used to inoculate embryonated eggs via the allantoic cavity^29^. Four days after inoculation, allantoic fluid was collected for the hemagglutination assay for determination of the EID_50_ titer^29^.

#### Confirmatory sequencing

All IAV-positive samples based on pan-IAV RTFRET-PCR were further verified by a PA gene-based IAV-generic PCR. The PCR was designed to target a variable region of PA gene of all IAV subtypes using the forward primer 5′-TTTGTGCGACAATGCTTCAATCC-3′ and the reverse primer 5′-TCGGACTGACGAAAGGAATCCCA-3′ which created a 575-bp PCR amplicon. The annealing temperature was 54 °C.

Amplification products of the pan-IAV RT FRET-PCR and the PA gene-based IAV-generic PCR were confirmed by electrophoresis of amplicons of 9 IAV subtypes through 1.5% MetaPhor agarose gels, purification using the QIAquick PCR Purification Kit (Qiagen, Valencia, CA, USA), and sequencing of both DNA strands using the forward and reverse primers (GenScript, Jiangsu, Nanjing, China).

### Screening of the IAV H9N2 subtype

A HA gene-based PCR specific for IAV H9N2^25^ was used to screen the IAV H9N2 in the IAV-positive samples determined by the one-step pan-IAV RT FRET-PCR. The PCR products were electrophoresed through 2% agarose gel (BIOWEST^®^, Hong Kong, China) and purified for automated DNA sequencing (GenScript).

### Statistical analysis

Comparison of the IAV positivity in oral-pharyngeal and cloacal swabs was analyzed by Chi-squared Test, and the comparison of the IAV copy number determined by PCR was performed by Student’s *t* Test. Differences were considered significant at P < 0.05.

## Additional Information

**How to cite this article**: Luan, L. *et al*. Detection of influenza A virus from live-bird market poultry swab samples in China by a pan-IAV, one-step reverse-transcription FRET-PCR. *Sci. Rep.* **6**, 30015; doi: 10.1038/srep30015 (2016).

## Supplementary Material

Supplementary Information

## Figures and Tables

**Table 1:** IAV isolates from Chinese LBMs used for validation and sensitivity determination of PCRs in this study.

IAV strain	IAV subtype	Detection limit	
		EID_50_	Pan-IAV PCR
A/Chicken/Jiangsu/W5-11/2014	H1N1	10^−6^	10^−7^
A/Chicken/Jiangsu/Ycd-2/2013	H3N3	10^−6^	10^−7^
A/Duck/Jiangsu/W14-5/2014	H4N6	10^−7^	10^−7^
A/Duck/Jiangsu/SY/2005	H5N1	10^−6^	10^−7^
A/Duck/Jiangsu/W7-5/2013	H6N1	10^−4^	10^−6^
A/Chicken/Jiangsu/ZJDT1-8/2014	H7N9	10^−8^	10^−8^
A/Chicken/Jiangsu/J10-3/2014	H9N2	10^−6^	10^−6^
A//Duck /Jiangsu/J3-5/2014	H10N8	10^−6^	10^−7^
A/Duck/Jiangsu/J14-3/2014	H11N2	10^−6^	10^−6^

**Table 2:** Prevalence of IAV for poultry in LBMs from 9 provinces of China by a pan-IAV PCR.

Regions	Chicken		Duck		Goose	Pigeon		Total	
	IAV	H9N2	IAV	H9N2	IAV	IAV	H9N2	IAV	H9N2
Jiangsu	70/155 (45.2)*	10/70 (14.3)**	3/31 (9.7)	0/3 (0.0)	0/9 (0.0)	4/46 (8.7)	0/4 (0.0)	77/241 (32.0)	10/70 (14.3)
Guangxi	39/130 (30.0)	39/39 (100)	3/10 (30.0)	3/3 (100)				42/140 (30.0)	42/42 (100)
Hebei	2/116 (1.7)	2/2 (100)	0/3 (0)			0/17 (0.0)		2/136 (1.5)	2/2 (100)
Shanxi	1/20 (5.0)	0/1 (0.0)						1/20 (5.0)	0/1(0.0)
Fujian	10/35 (28.6)	10/10 (100)	1/33 (3.0)	1/1 (100)				11/68 (16.2)	11/11 (100)
Shaanxi	5/70 (7.1)	0/5 (0.0)						5/70 (7.1)	0/5(0.0)
Hunan	1/70 (1.4)	1/1 (100)						1/70 (1.4)	1/1 (100)
Yunnan	22/70 (31.4)	22/22 (100)						22/70 (31.4)	22/22 (100)
Hainan	8/70 (11.4)	8/8 (100)						8/70 (11.4)	8/8 (100)
Total	158/736 (21.5)	92/158 (58.2)	7/77 (9.1)	4/7 (57.1)	0/9 (0.0)	4/63 (6.3)	0/4 (0.0)	169/885 (19.1)	96/169 (56.8)

*Indicates the IAV positivity determined by pan-IAV PCR shown as number of positive poultry/total poultry (positivity in percentage).

**Indicates the number of H9N2 among all IAV-positive ones.

***IAV was not detected in the poultry swabs from the following 15 provinces: Sichuan, Hubei, Xinjiang, Guangdong, Henan, Shandong, Anhui, Tibet, Jilin, Liaoning, Gansu, Jiangxi, Zhejiang, Inner Mongolia, and Shanghai.

**Table 3:** IAV subtypes detectable by the pan-IAV RT-FRET-PCR.

	N1	N2	N3	N4	N5	N6	N7	N8	N9
H1	x*	x	x	x	x	x	x	x	x
H2	x	x	x	x	x	x	x	x	x
H3	x	x	x	x	x	x	x	x	x
H4	x	x	x	x	x	x	x	x	x
H5	x	x	x	x	x	x	x	x	x
H6	x	x	x	x	x	x	x	x	x
H7	x	x	x	x	x	x	x	x	x
H8	x	x	x	x	—**	x	—	x	—
H9	x	x	x	x	x	x	x	x	x
H10	x	x	x	x	x	x	x	x	x
H11	x	x	x	x	x	x	x	x	x
H12	x	x	x	x	x	x	x	x	x
H13	—	x	x	—	—	x	—	x	x
H14	—	x	x	—	x	x	—	x	—
H15	—	—	—	x	x	x	x	x	x
H16	—	—	x	—	—	—	—	—	—

^*^Each of the four primers/probes was individually BLAST-searched against the IAV subtype in GenBank (Table S1), and subtypes with sequences that matched all PCR oligonucleotides were assumed detectable.

^**^“—” denotes that the universal IAV PCR either cannot amplify or detect the designated IAV subtype, or this IAV subtype does not exist.
