# Supplementary material for: Detection of influenza A virus from live-bird market poultry swab samples in China by a pan-IAV, one-step reverse-transcription FRET-PCR
Source: Sci Rep. 2016 Jul 22;6:30015. doi: 10.1038/srep30015 (PMC4957103; doi:10.1038/srep30015)
Supplement: Supplementary Information [file srep30015-s1.pdf]

Detection of influenza A virus from live-bird market poultry swab samples in China by a pan-IAV, one-step reverse-transcription FRET-PCR

Lu Luan[1], Zhihao Sun[1], Bernhard Kaltenboeck[2], Daxin Peng[1], Xiulong Xu[1], Jianqiang Ye[1], Jing Li[1], Weina Guo[1], Ke Huang[1], Min Li[1], Chengming Wang[1*]

Table S1. Strain/isolate numbers of IAV serotypes matching the pan-IAV primers/probes.

IAV	Forward primer	Reverse primer	6-FAM probe	LCRed-640 probe	Minimum strains/isolates detectable
**H1N1**	7908	7284	7831	7516	7284
**H1N2**	1426	1444	1545	1526	1426
**H1N3**	67	120	37	99	37
**H1N4**	15	24	12	31	12
**H1N5**	26	43	18	30	18
**H1N6**	47	109	54	75	47
**H1N7**	11	22	11	11	11
**H1N8**	45	59	30	46	30
**H1N9**	84	126	79	141	79
**H2N1**	153	166	92	177	92
**H2N2**	238	200	187	242	187
**H2N3**	201	173	155	176	155
**H2N4**	8	8	7	11	8
**H2N5**	42	84	44	70	42
**H2N6**	8	17	9	15	8
**H2N7**	73	88	55	92	55
**H2N8**	36	41	22	43	22
**H2N9**	151	240	145	179	145
**H3N1**	198	218	125	235	125
**H3N2**	7827	7068	7726	7279	7068
**H3N3**	50	58	35	64	35
**H3N4**	7	12	4	7	4
**H3N5**	35	74	37	78	35
**H3N6**	271	193	235	264	193
**H3N7**	23	28	16	29	16
**H3N8**	978	811	1016	918	811
**H3N9**	17	22	14	26	14
**H4N1**	42	46	25	42	25
**H4N2**	192	149	119	174	119
**H4N3**	65	81	48	71	48
**H4N4**	24	38	16	33	16
**H4N5**	41	61	22	50	22
**H4N6**	698	666	712	809	666
**H4N7**	25	34	16	33	16
**H4N8**	153	150	136	216	136
**H4N9**	85	99	57	90	57
**H5N1**	2274	2071	2061	2195	2061
**H5N2**	593	559	582	553	553
**H5N3**	101	317	187	271	101

**H5N4**	14	16	16	16	14
**H5N5**	85	120	47	129	47
**H5N6**	89	546	176	292	89
**H5N7**	25	31	16	29	16
**H5N8**	101	99	112	101	99
**H5N9**	62	72	40	69	40
**H6N1**	375	347	332	326	326
**H6N2**	449	420	433	426	420
**H6N3**	33	50	48	49	33
**H6N4**	44	55	27	54	27
**H6N5**	141	253	115	268	115
**H6N6**	185	156	170	179	156
**H6N7**	10	11	7	13	7
**H6N8**	168	160	159	160	159
**H6N9**	31	32	24	40	24
**H7N1**	142	143	148	225	142
**H7N2**	315	274	315	314	274
**H7N3**	308	346	301	311	301
**H7N4**	55	89	43	68	43
**H7N5**	13	9	8	13	8
**H7N6**	80	113	79	128	79
**H7N7**	200	183	180	214	180
**H7N8**	59	51	43	59	43
**H7N9**	450	453	455	450	450
**H8N1**	3	5	1	3	1
**H8N2**	6	11	5	10	5
**H8N3**	3	4	2	5	2
**H8N4**	97	102	96	99	96
**H8N5**	0	1	0	0	0
**H8N6**	3	3	3	3	3
**H8N7**	0	2	0	0	0
**H8N8**	3	4	2	4	2
**H9N1**	50	88	46	69	46
**H9N2**	1590	1459	1613	1204	1204
**H9N3**	5	22	8	8	5
**H9N4**	3	8	6	5	3
**H9N5**	26	73	31	62	26
**H9N6**	9	19	7	17	7
**H9N7**	15	21	18	15	15
**H9N8**	19	19	12	15	12
**H9N9**	37	53	35	52	35
**H10N1**	61	106	53	71	53
**H10N2**	52	98	49	92	49
**H10N3**	139	175	112	191	112
**H10N4**	77	176	89	109	77

**H10N5**	47	90	40	61	40
**H10N6**	77	157	75	80	75
**H10N7**	249	327	254	262	249
**H10N8**	216	290	144	193	144
**H10N9**	38	77	41	57	38
**H11N1**	73	115	65	107	65
**H11N2**	90	372	168	378	90
**H11N3**	58	91	43	86	43
**H11N4**	2	4	2	2	2
**H11N5**	11	20	9	12	9
**H11N6**	35	60	29	43	29
**H11N7**	16	26	13	14	13
**H11N8**	49	63	30	51	30
**H11N9**	257	567	278	263	257
**H12N1**	23	25	14	19	14
**H12N2**	11	15	8	11	8
**H12N3**	17	24	12	16	12
**H12N4**	23	39	18	36	18
**H12N5**	96	112	201	94	94
**H12N6**	13	23	11	18	11
**H12N7**	12	17	10	18	10
**H12N8**	10	16	10	14	10
**H12N9**	9	13	9	14	9
**H13N1**	2	0	0	1	0
**H13N2**	84	75	37	104	37
**H13N3**	5	6	5	7	5
**H13N4**	1	1	0	1	0
**H13N6**	130	181	111	222	111
**H13N8**	28	36	15	27	15
**H13N9**	47	76	22	37	22
**H14N2**	3	9	3	11	3
**H14N3**	3	3	4	4	3
**H14N5**	12	18	9	16	9
**H14N6**	22	20	11	19	11
**H14N8**	9	7	4	5	4
**H15N2**	0	1	1	2	0
**H15N4**	4	5	3	4	3
**H15N6**	1	3	2	5	1
**H15N7**	4	5	3	4	3
**H15N8**	2	3	3	4	2
**H15N9**	6	16	9	19	6
**H16N3**	105	150	76	138	76
**H16N9**	1	2	1	3	1
**Total**	**31776**	**32641**	**30807**	**32366**	**28206**

9

10

Table S2. IAV serovars used for Clustal Alignment Algorithm and designing of primers and probes in this study.

H subtype	N subtype and gene accession number of IAVs
**H1**	N1: CY103734; N2: CY133291, N3: CY134400; N9: CY167119
**H2**	N1: CY191028, N2: CY121928; N3: FJ357078; N7: CY157543, N9: CY184254
**H3**	N1: CY132294; N2: JX235984; N6: CY004305; N8: AY633341
**H4**	N2: KM244117; N6: CY101572, N8: AF250492, N9: AB916686
**H5**	N1: FJ434375; N2: AY300953; N3: CY167193; N4: CY096683; N7: CY094438; N8: GQ923574; N9: CY133786
**H6**	N1: KJ568060; N2: KJ0246008; N5: KM244095; N6: KJ200910; N8: KJ568324
**H7**	N1: KJ567948; N3: CY081333; N4: CY133778; N6: CY125374; N7: CY167233; N8: CY132510; N9: KF150611
**H8**	N1: CY166785; N3: CY076022; N4: CY064122
**H9**	N1: CY181338; N2: GU051520; N4: GU051538; N7: KF591858; N8: DQ021759; N9: KJ938653
**H10**	N2: CY166395; N4: CY190332; N7: CY005232
**H11**	N1: CY126705; N2: LC042068; N6: CY120527; N9: CY181274
**H12**	N1: JN982514; N2: CY166227; N4: CY133358; N5: GU050870
**H13**	N2: KR087567; N6: CY185674; N8: CY185634
**H14**	N2: CY167268; N5: GU052253; N6: KC110606; N8: CY133554
**H15**	N6: GU052261; N7: KP087871; N9: CY185888
**H16**	N3: AB937724; N9: KC845030
